# Scurvy

**Published:** 1873-01

**Authors:** J. M. DaCosta


### ARTICLE VIII.

### *Scurvy.*

The first case to which I shall call attention is Alexander F., a native of Switzerland, aged forty-two years. He has been three years in this country, principally employed as a clerk. He has never suffered from venereal disease nor rheumatism, and has been a "total abstinence" man. He suffered from the small-pox twenty-three years ago, and five years subsequently he had great vomiting of blood. Twelve years ago he had another such attack, which was followed by yet another six days previous to his admission into this hospital, which was October 28th, of this year.

We have thus several attacks of vomiting of blood to record, also epistaxis, but of late years his nose does not bleed oftener than two or three times a year. His parents are aged and healthy. In no respect can be traced any hereditary cause for this frequent bleeding. For the last three years he has passed urine frequently and in small quantities.

In 1870 his gums became swollen and spongy, tender to the touch, and bleeding when the teeth were pressed together. His hair fell out, but he suffered from no other sickness, neither had he taken any mercurial. He was at that time traveling in Missouri, and his food was of that varied kind generally supplied by hotels. He has never been in want; never worked in lead. Before he came here he lived in Baltimore, where he lived comfortably, having employment as clerk in a coal yard. He suffered, however, from a certain amount of mental worriment, and his sleep was impaired. With this last named exception he was well. Until two months ago his gums were swollen and bled very easily, and although his appetite was good he could eat no solid food.

Five days prior to his admission into the hospital he found that walking caused him great pain, and one morning he perceived a large spot, or ecchymosis, on his left leg just above the ankle. He had received no injury or fall. He came to Philadelphia, and on his admission to the hospital his face was sallow and puffy; his gums spongy and ulcerated; mouth dry (no signs of salivation ;) appetite poor; sleep bad and bowels constipated. He had no thirst nor headache. He urinated four or five times daily, and as many times at night. His urine was acid in its reaction, S. g. 1014, but did not contain albumen.

He had pain in both legs below the knee; the left leg was swollen and shiney, and discolored and yellowish from the knee down. On each side of his ankle were large patches of ecchymoses. He was put to bed, and at his own request the *limb* was put in a fracture box and bathed with cold water, as being more easy and acceptable to him. He was also directed to have a mouth wash of chlorate of potash; and in this condition I found him a few days ago.

He is now before you and his case speaks for itself. His gums are still spongy, and it hurts him to press his teeth together. His face is still pale and so are his conjunctivæ; but his face looks better than on admission. His heart beats rapidly, his pulse is sharp and distinct. There is a faint systolic blood murmur at the base. His tongue is pale, not coated, and he suffers from no abdominal pain. The leg is still discolored, as from a bruise. It is not so much so, however, as on his admission. Purple spots are still effused underneath the skin, and he cannot walk on account of the pain. He is at present taking with relief the mineral acids and iron, with chlorate of potassa, locally, for the mouth.

℞ Acid nitro muriat gtt. x.

*t. d.* in water, after meals.

His diet is varied and liberal, with a plentiful supply of fresh vegetables and lemonade.

This is a curious illustration of scurvy happening without any apparent cause. He has not lived a life of want or privation, and yet, owing to some curious condition of his blood, he presents about as marked a case as you would find on any ship. Scurvy is usually, and very rightly, attributed to want of fresh vegetable diet; and the exclusive use of salt meats will predispose to scurvy. Persons living exclusively upon fresh meat would also become scorbutic, although not as soon as when the salt meats are solely partaken of.

I cannot trace any such cause here, as his diet was varied, and he partook of vegetable food. There seems to be, looking back upon his history, some curious condition as to the state of the blood, no matter what diet he was upon.

We find among army troops, that as long as their *morale* is kept up, and their cheerfulness maintained, so long they will escape scurvy, or have it very lightly. But when there is dull routine, and the men are mentally distressed, it will have a good deal to do with the developing of this disorder.

To this patient's mental distress I attribute, in part, his last attack, although this scorbutic blood taint seems to have pursued him all his life. The symptoms grouped here are typical; the condition of the gums, the pale, smooth tongue and the anæmic eye, all belong to the history of the disease. The spots near the joints are also diagnostic of scorbutic patients. These are the cause of this man's trouble in walking; and we often find them giving rise to apparent malformation, as for instance the contraction of certain groups of muscles.

I have spoken to you of a scorbutic diathesis, and of an altered state of the blood. What is the state of the blood in these cases? We find it lessened in density and fibrine; but accurate chemical investigation is yet wanting to develop the full extent of change that occurs. Scurvy was at one time attributed to a want of the potash salts, but this doctrine cannot be maintained, and the most accurate observations prove it to be fallacious

Pains in the limbs, as we have in this case, and back, are prominent symptoms of scurvy. During the late war I saw many cases in which pain in the back, with the exception of the state of the gums, was the only distinctive symptom. We must remember this manifestation of a scorbutic state of the symptom; otherwise we might judge our patient simply to suffer from muscular pains, or to be a malingerer. Scurvy, in private practice, is so rare a disease, that some physicians have never seen it.

This man is rapidly recovering. His treatment will be continued, laying great stress on his vegetable diet and his lemonade.—*Clinic of J. M. DaCosta, M. D. Reported by R. M. Townsend, M. D., in Med. and Surg. Reporter.*

